# The Genus *Spinopygina* gen. nov. (Diptera, Sciaridae) from Western North America: Preliminary Molecular Phylogeny and Description of Seven New Species

**DOI:** 10.3390/insects14020173

**Published:** 2023-02-09

**Authors:** Pekka Vilkamaa, Nikola Burdíková, Jan Ševčík

**Affiliations:** 1 Finnish Museum of Natural History, Zoology Unit, University of Helsinki, P.O. Box 17, FI-00014 Helsinki, Finland; 2 Department of Biology and Ecology, Faculty of Science, University of Ostrava, Chittussiho 10, CZ-710 00 Ostrava, Czech Republic; 3 Silesian Museum, Nádražní Okruh 31, CZ-74601 Opava, Czech Republic

**Keywords:** Sciaridae, black-winged fungus gnats, new taxa, phylogeny, Nearctic Region

## Abstract

**Simple Summary:**

Black-winged fungus gnats (family Sciaridae) are one of the most species-rich groups of flies (Diptera), with about 3000 described species worldwide. Their taxonomy is notoriously challenging, as many genera are difficult to delimit and species identification relies almost exclusively on the male genitalia. Existing phylogenetic hypotheses based on DNA sequences are often contradictory, presumably due to low taxon or gene sampling. In this paper, a new multigene phylogenetic analysis is presented to support the concept and monophyly of a new genus, containing eight species, seven of them being described as new to science.

**Abstract:**

The genus *Spinopygina* gen. nov. (type species *Camptochaeta uniceps* Hippa & Vilkamaa, 1994) from western North America is described. The genus includes the following eight species: *Spinopygina acerfalx* sp. nov.; *S. aurifera* sp. nov.; *S. camura* sp. nov.; *S. edura* sp. nov.; *S. peltata* sp. nov.; *S. plena* sp. nov.; *S. quadracantha* sp. nov.; and *S. uniceps* (Hippa & Vilkamaa, 1994) comb. nov., transferred from *Corynoptera* Winnertz. The new species are described and *Spinopygina uniceps* is re-diagnosed. The species are keyed and illustrated. In the maximum-likelihood phylogenetic hypothesis based on four gene fragments (28S, 18S, 16S and COI), *Spinopygina* gen. nov. appears as the sister group of *Claustropyga* Hippa, Vilkamaa & Mohrig, 2003. In the same analysis, a remarkable, undescribed species is placed within *Camptochaeta* Hippa & Vilkamaa clade.

## 1. Introduction

Sciaridae (black-winged fungus gnats) is one of the little studied terrestrial Dipteran families with about 3000 described species in about 100 genera and subgenera, occurring in all continents [1]. Most species live in shady forested and moist habitats. Larvae are predominantly saprophagous and live in soil and litter or under bark of dead wood. The taxonomy is notoriously challenging, as many genera are difficult to delimit and are rich in small and rather similar species. The species identification relies almost exclusively on the male hypopygium, and in most genera the females are unidentifiable. Some trials to reconstruct phylogenies extensively over the family or focused on a limited number of taxa have been made, either using morphological characters [1,2,3,4,5,6,7,8] or using molecular markers [9,10,11,12]. Often the hypotheses obtained have been contradictory, presumably due to variable choice of morphological characters, insufficient number of molecular characters used or because of low taxon sampling. The paper by Shin et al. [9] is the most comprehensive of the molecular analyses, but it misses many crucial genera from the Holarctic region and lacks almost completely the extra-Holarctic taxa in its taxon sampling. Shin et al. [9] divided the family into three subfamilies, and later [10] named a fourth, but the large and morphologically very diverse group of genera (the ‘*Pseudolycoriella* group’) appeared non-monophyletic and was not ranked as a subfamily.

Of the Holarctic fauna of Sciaridae, the Nearctic region is much less studied than the Palaearctic fauna. Mohrig et al. [13] in their revision listed 166 valid species from the Nearctic region (excluding Greenland), and subsequent authors [14,15,16,17,18,19,20,21,22,23,24,25,26] have added 70 new species,making a total of 236 known species in the Nearctic to date. The number of known Palaearctic species is much higher, about 2000. Based on the COI (barcode) gene, Hebert et al. [27] counted 2200 different BINs for Sciaridae from Canada, but proper taxonomic work is waiting to identify and name the taxa. Other biogeographic regions are even less studied than the Holarctic, but studies made on different genera indicate that the taxonomic richness of Sciaridae in the tropics is enormous [2,28,29,30,31,32,33,34,35].

Hippa and Vilkamaa [36] described *Camptochaeta uniceps* from Canada (British Columbia) and included the species in their new genus *Camptochaeta* because it has the lambda-shaped basomedial sclerotization in the gonostylus, regarded as a synapomorphy of *Camptochaeta*. Subsequently, Menzel and Mohrig [1] in their revision of the Palaearctic Sciaridae did not accept the concept of *Camptochaeta,* but transferred two of the included species into *Keilbachia* Mohrig, 1987 and all other species lacking the apical tooth of the gonostylus into the *Corynoptera parvula* group and into the *C. spinifera* group of the large but obviously non-monophyletic *Corynoptera* Winnertz, 1867. Later, the Nearctic *Camptochaeta uniceps* Hippa &Vilkamaa, 1994 was transferred to the *Corynoptera spinifera* group by Mohrig et al. [13].

Here, in this study, we report on several species undoubtedly related to C. uniceps from western North America. We were able to study the species complex in more detail, describe the new species and consider the phylogenetic position and the taxonomic status of the group. Simultaneously, the taxonomic placement of a new species of Sciaridae (*Camptochaeta* sp., voucher SCI87) with an unusual tegmen was searched for.

## 2. Materials and Methods

### 2.1. Morphological Methods

The material originated from Malaise trap samples, and all specimens were detected and picked out from unsorted sciarid or insect material stored in ethanol. The holotype of *Camptochaeta uniceps* was obtained from Canadian National Collection, Ottawa, Canada (CNC) and a paratype from Royal British Columbia Museum, Victoria, Canada (RBCM), the other paratypes having been deposited in Zoological Museum, Finnish Museum of Natural History. The specimens were mounted on microscope slides in Euparal, after dehydrating them in absolute ethanol. Only males were studied: the females are unknown. The terminology and methods of measuring structures follow Hippa and Vilkamaa [28] and Hippa et al. [3,37]. The photographs of the slide-mounted specimens were taken with a Leica MC170 HD camera mounted on Leica DM 4000 B LED research microscope. The habitus of *Spinopygina uniceps* was photographed with Canon EOS 5DS digital SLR camera with a Canon MP-E 65 mm macro lens. The Figures were processed with Photoshop version CS5, CorelDraw2017 and CorelPhotopaint2017. The type material of the new species is deposited in Zoological Museum, Finnish Museum of Natural History, Helsinki, Finland (MZH). The specimen data of the MZH specimens will be added into the specimen database of the Finnish Museum of Natural History (Luomus) and will be found with the specimen codes https://luomus/GE.####.

This published work and the nomenclatural acts it contains have been registered in ZooBank, the online registration system for the ICZN. The LSID for this publication is: urn:lsid:zoobank.org:pub:9962DD66-13CC-4191-B6FF-0A7DD1934F75.

### 2.2. Molecular Methods

For the ingroup, representatives of taxa from the three subfamilies of Sciaridae were chosen as well as selected terminals from the non-monophyletic ‘*Pseudolycoriella* group’ of genera proposed by Shin et al. [9,10]. From the latter group, terminals were chosen to adequately cover the morphological diversity of the group.

The specimens used for DNA analysis (Table 1) were alcohol-preserved (70% to 99.9% ethanol). The DNA was extracted using NucleoSpin Tissue Kit (Macherey-Nagel, Düren, Germany) following manufacturers’ protocols. PCRs were performed using primers listed by Shin et al. [9].

All amplified products were purified using Gel/PCR DNA Fragments Extraction Kit (Geneaid, New Taipei City, Taiwan) and subsequently, the samples were sequenced by Eurofins (Hamburg, Germany). All sequences were assembled, manually inspected, and primers trimmed in SeqTrace [38]. New sequences were deposited in the GenBank database, with their accession numbers listed in Table 1.

All genes were aligned using MAFFT version 7 [39] on the MAFFT server (http://mafft.cbrc.jp/alignment/server/). To remove all unreliably aligned regions, the GBLOCKS 0.91b program [40] was used (http://phylogeny.lirmm.fr/phylo_cgi/one_task.cgi?task_type=gblocks) with conditions set as follows: allow smaller blocks, allow gap positions within the final blocks, allow less strict flanking positions and do not allow many contiguous nonconserved positions. The final molecular dataset consists of 4287 bp, and the lengths of individual alignments were: 18S = 1843 bp, 28S = 1308 bp, 16S = 478 bp, COI = 658 bp.

The final concatenated dataset was partitioned by gene and codon position and subsequently analysed using ML method with IQtree [41]. Best-fitting substitution models were chosen automatically by the IQ-TREE software (GTR+F+I+G4: 16S, TPM3u+F+I+G4: 18S, TVM+F+I+G4:28S, TIM3+F+G4: COI_1, TIM2+F+I+G4: COI_2, TIM3+F+I+G4: COI_3), without free-rate heterogeneity. Branch supports were evaluated using 1000 ultrafast bootstrap [42]. All other settings were left as default. The resulting phylogenetic tree (consensus tree) was visualized using Interactive Tree of Life (iTOL; [43]).

## 3. Results

### 3.1. Desription of the New Genus

***Spinopygina*** **gen. nov.**

Type species: *Camptochaeta uniceps* Hippa & Vilkamaa, 1994.

LSID urn:lsid:zoobank.org:act: DBFE5207-337A-4817-B955-14847009D23B.

**Etymology.** The name is formed from the Latin word spine, thorn, and the Greek word pyge, rump, referring to the outstanding megasetae of the gonostylus of the species.

**Diagnosis.** Small to medium sized Sciaridae, wing length 1.6–2.2 mm. Antenna long, flagellomeres with long setae and necks. Mouth parts small, maxillary palpus with 2 segments, 2nd segment much reduced. Anal lobe of wing small, halter with long stalk. Body setosity dark, long and strong. Legs long and slender, fore tibial organ poorly differentiated. Intergonocoxal area long or moderate, without medial lobe. Gonocoxae united medially, setae at medial margins short. Gonostylus impressed or deeply excavated medially, with lambda-shaped basomedial sclerotization; with strong megasetae with basal bodies, including (in most species) strongly procurved basal megaseta; without apical tooth. Tegmen modified, with flat apicolateral parts, without detectable aedeagal teeth.

**Figure 1:**
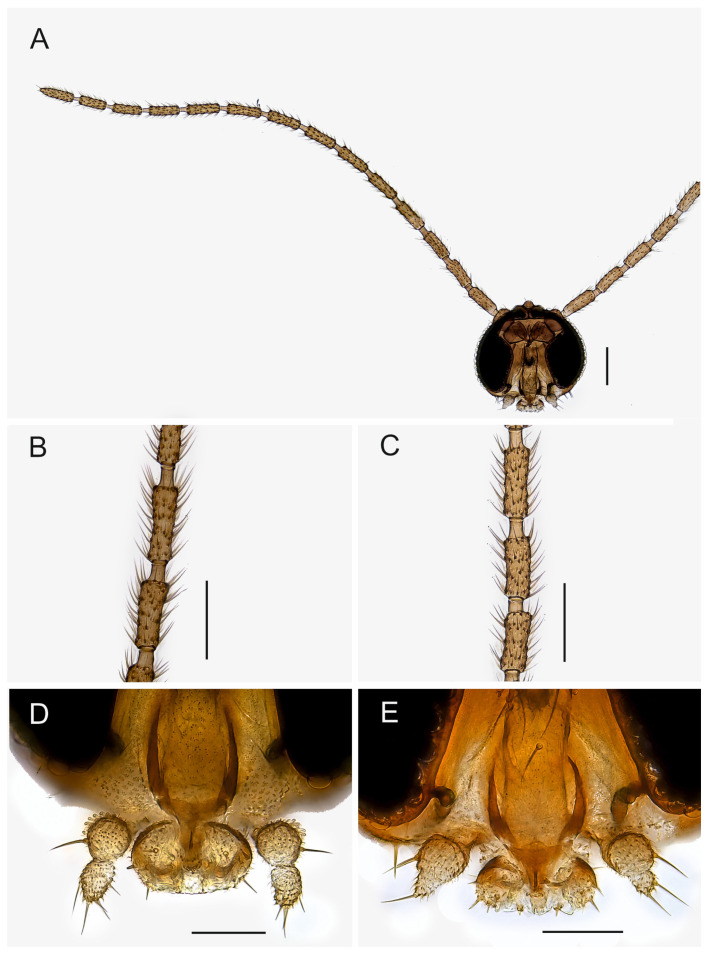
(**A**). Head, frontal view (holotype MZH GE.1941 of *Spinopygina plena* sp. nov.). (**B**). Antennal flagellomeres 3–4, frontal view (paratype MZH GE.1940 of *S. peltata* sp. nov.). (**C**). Antennal flagellomeres 2–4, frontal view (paratype MZH GE.1936 of *S. camura* sp. nov.). (**D**). Mouth parts, frontal view (holotype MZH GE.1932 of *S. aurifera* sp. nov.). (**E**). Mouth parts, frontal view, (holotype MZH GE.1941 of *S. plena* sp. nov.). Scale bars for (**A**–**C**) = 0.1 mm, for (**D**,**E**) = 0.05 mm.

**Description.** Head (Figure 1). Normal, roundish. Eye bridge 1 to 2 facets wide. Anterior vertex non-setose. Face setose. Clypeus with 1–2 setae or non-setose. Antennal scape and pedicel normal. Antennal flagellum long, with 14 flagellomeres, bodies of flagellomeres subcylindrical, with smooth surface, body of 4th flagellomere 2.25–3.20× as long as subapically wide, necks longer than wide. Flagellomeral vestiture rather sparse, setae longer than width of flagellomeres. Mouth parts small. Maxillary palpus with 2 segments, 2nd segment strongly reduced, variably between species and even specimens; 1st segment with 1, rarely with 2 sharp setae; with long sparse sensilla dorsally.

**Figure 2:**
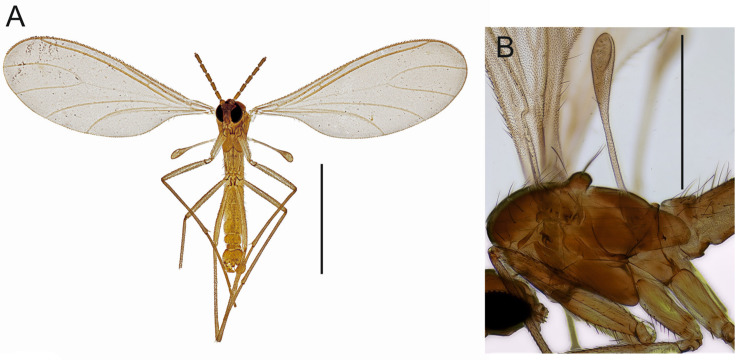
(**A**). Habitus, ventral view, specimen MZH GE.1969 of *Spinopygina uniceps* (Hippa & Vilkamaa; 1994). (**B**). Thorax, lateral view (paratype MZH GE.1931 of *S. acerfalx* sp. nov.). Scale bars for (**A**) = 1.0 mm, for (**B**) = 0.50 mm.

Thorax (Figure 2). Brown; setae dark. Acrostichal setae few anteriorly, dorsocentral and lateral setae in indistinct rows of a few long and strong and short and fine setae. Scutellum with 2 long and strong setae and a few short and fine setae. Posterior pronotum non-setose. Anterior pronotum and prothoracic episternum with a few setae, other pleural sclerites non-setose. Mesothoracic katepisternum high with oblique anterior margin.

**Figure 3:**
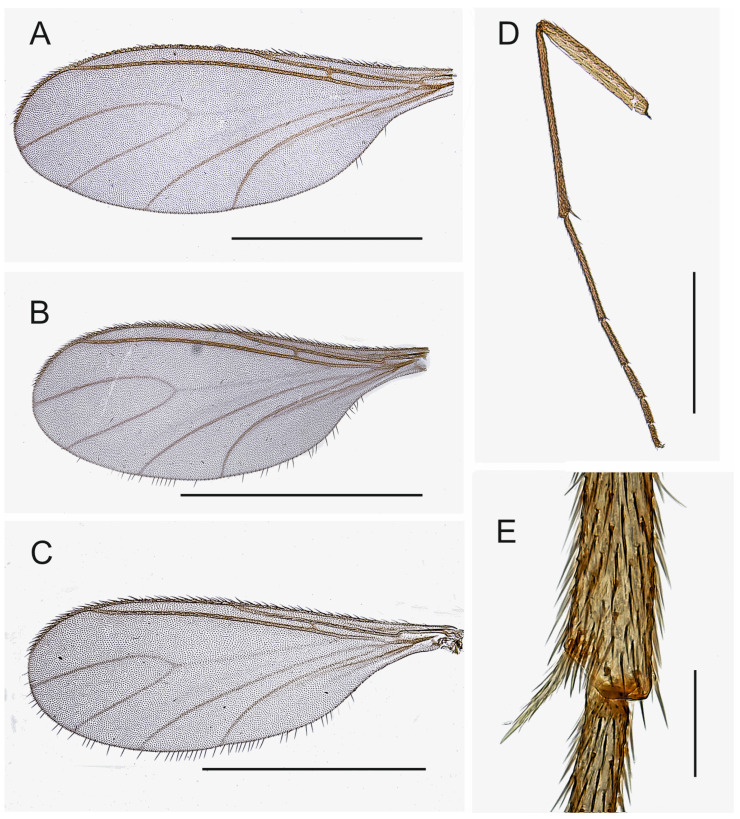
(**A**–**C**). Wings, ventral view. (**A**). Paratype MZH GE.1929 of *Spinopygina acerfalx* sp. nov. (**B**). Paratype MZH GE.1933 of *S. aurifera* sp. nov. (**C**). Paratype MZH GE.1981 of *S. plena* sp. nov. (**D**). Foreleg, prolateral view (holotype MZH GE.1938 of *S. peltata* sp. nov.) (**E**). Apical part of fore tibia, prolateral view (paratype MZH GE.1942 of *S. plena* sp. nov. Scale bars for (**A**–**C**) = 1.0 mm, for (**D**) = 0.5 mm, for (**E**) = 0.05 mm.

Wing (Figure 3A–C). Fumose. Length 1.6–2.2 mm. Anal lobe small. Veins distinct, except stM. Membrane non-setose, veins C, R and R1 setose, R5 with only dorsal setae, bM, r-m, stM, M and stCuA non-setose. c/w 0.70–0.90, R1/R 0.70–1.15, R1 joining C well before level of base of M-fork. Halter yellow, with long stalk.

Legs (Figure 3D,E). Yellow or pale brown, long, femora slender; setae dark; tibial spurs 1:2:2, fore tibial spur as long as tibial width or slightly longer, middle and hind left and right tibial spurs subequal in size, longer than tibial width. Fore tibia without spinose setae, middle tibia rarely with 1 spinose seta, hind tibia with dorsal row of strong spinose setae. The retrolateral apical setae of hind tibia fine. Fore tibial organ not impressed or proximally bordered, with small patch of a few setae. Tarsi unmodified, long, length of fore basitarsomere/length of fore tibia 0.47–0.63. Tarsal claws unmodified, without teeth.

**Figure 4:**
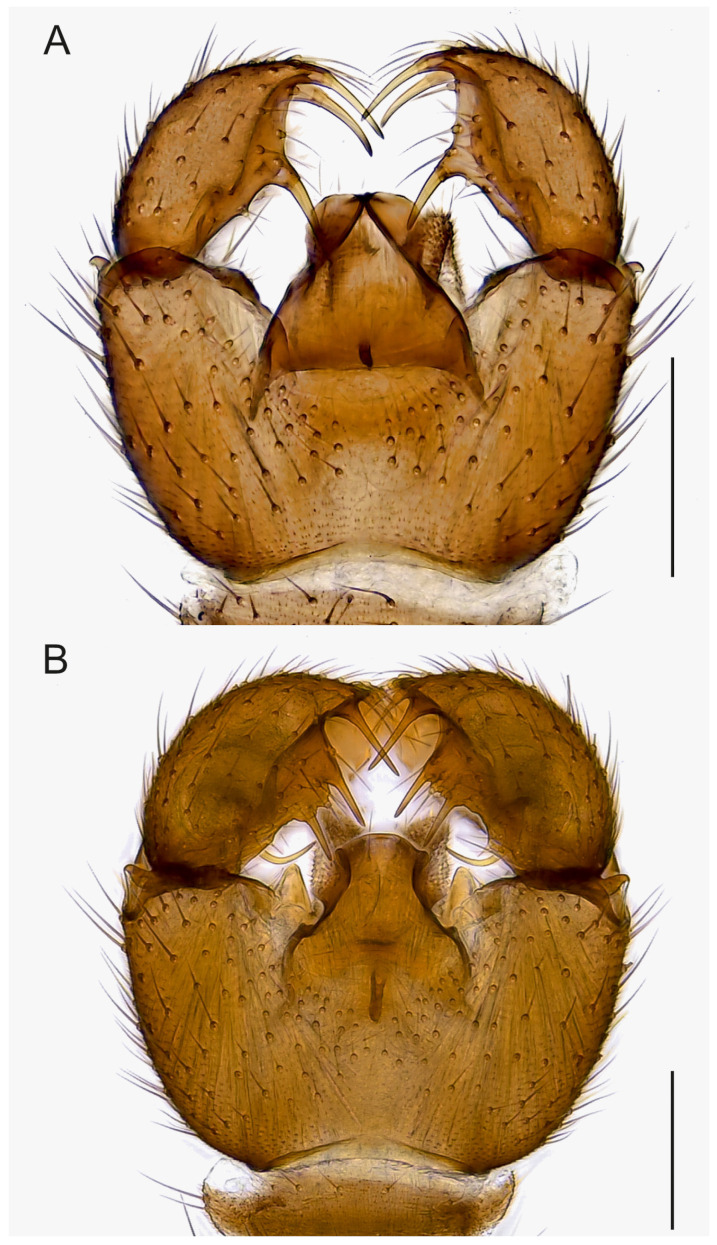
Hypopygia, ventral view. (**A**). Holotype MZH GE.1928 of *Spinopygina acerfalx* sp. nov. (**B**). Holotype MZH GE.1932 of *S. aurifera* sp. nov. Scale bars = 0.1 mm.

**Figure 5:**
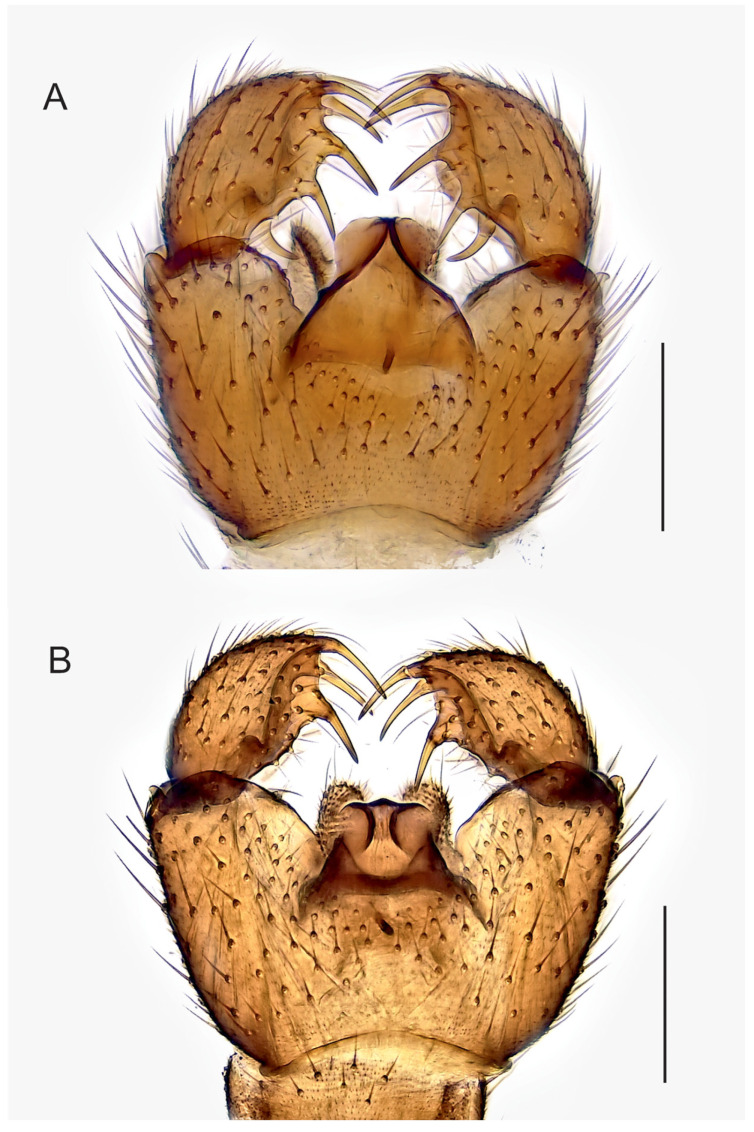
Hypopygia, ventral view. (**A**). Holotype MZH GE.1935 of *Spinopygina camura* sp. nov. (**B**). Holotype MZH GE.1937 of *S. edura* sp. nov. Scale bars = 0.1 mm.

**Figure 6:**
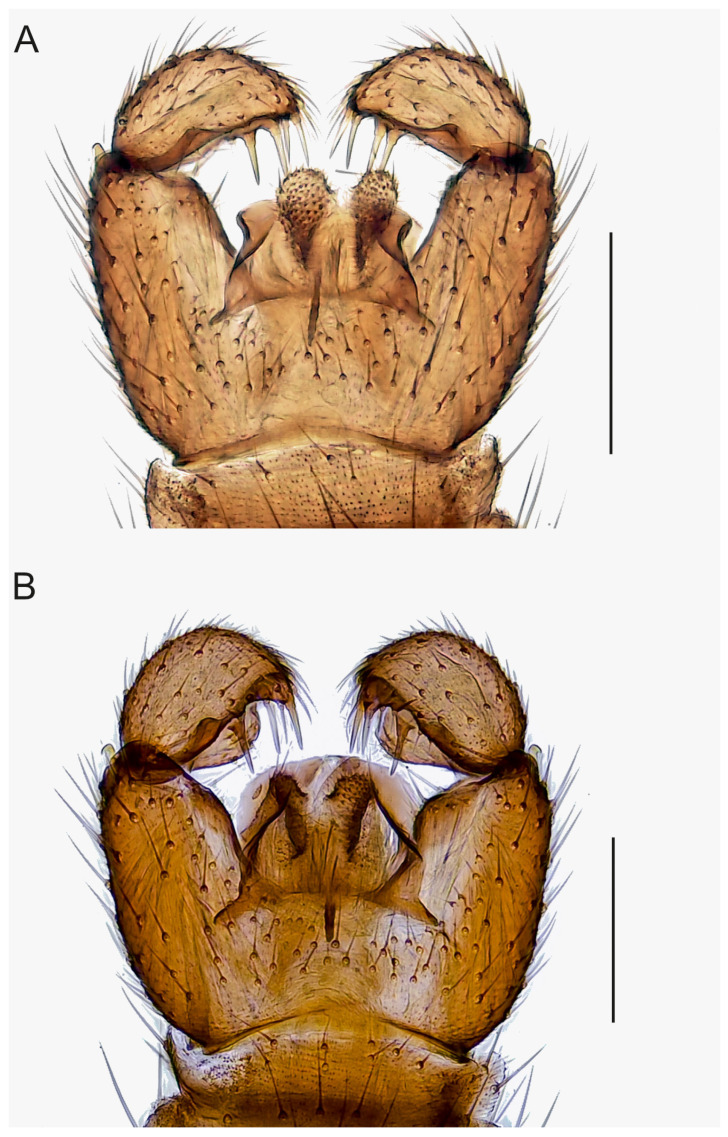
Hypopygia, ventral view. (**A**). Holotype MZH GE.1938 of *Spinopygina peltata* sp. nov. (**B**). Holotype MZH GE.1941 of *S. plena* sp. nov. Scale bars = 0.1 mm.

**Figure 7:**
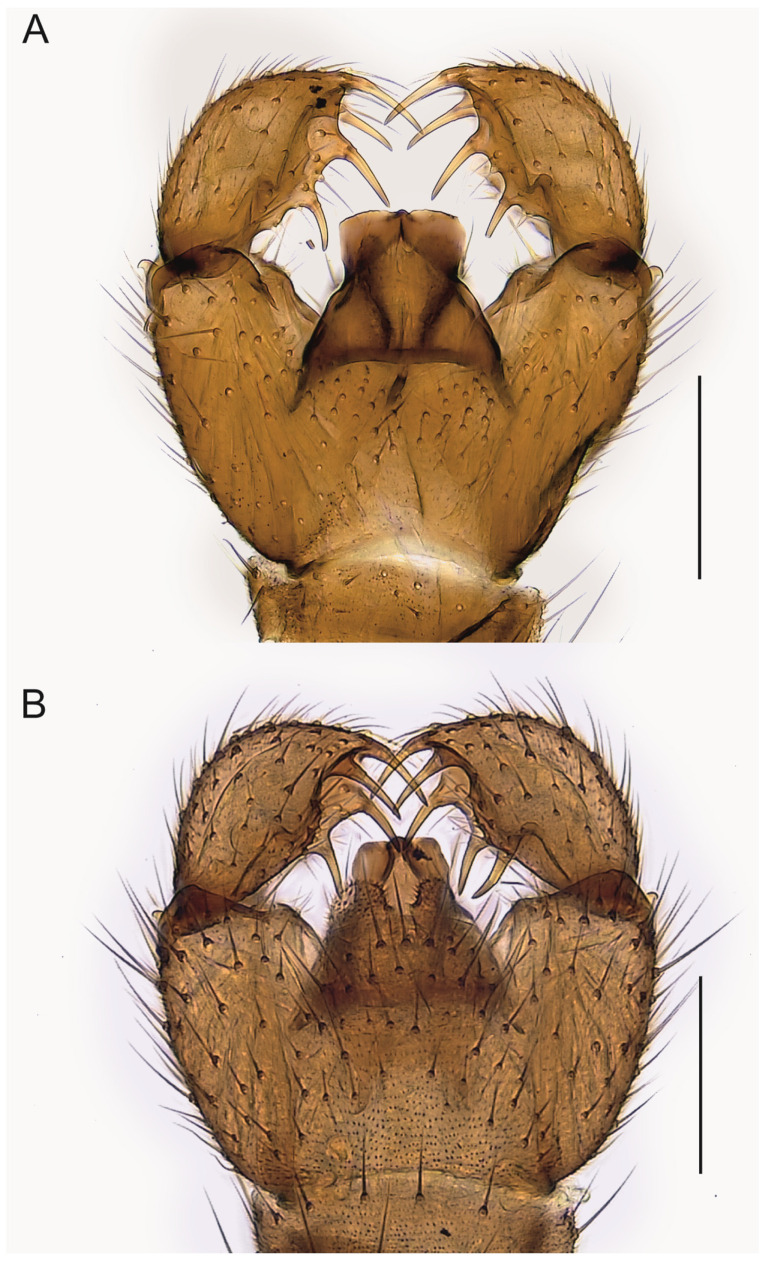
Hypopygium of *Spinopygina quadracantha* sp. nov. (**A**). Ventral view (holotype MZH GE.1944). (**B**). Dorsal view (paratype MZH GE.1956). Scale bars = 0.1 mm.

**Figure 8:**
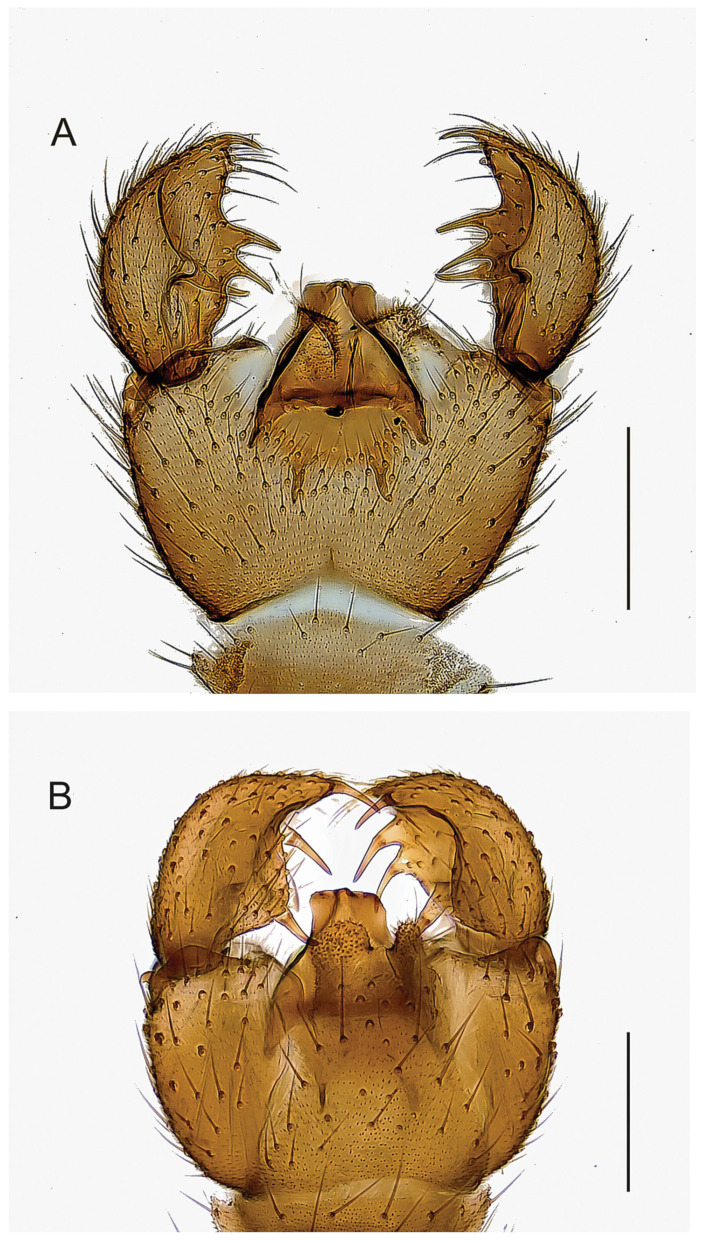
Hypopygium of *Spinopygina uniceps* (Hippa & Vilkamaa, 1994). (**A**). Ventral view (holotype CNC). (**B**). Dorsal view (specimen MZH GE.1962). Scale bars = 0.1 mm.

**Figure 9:**
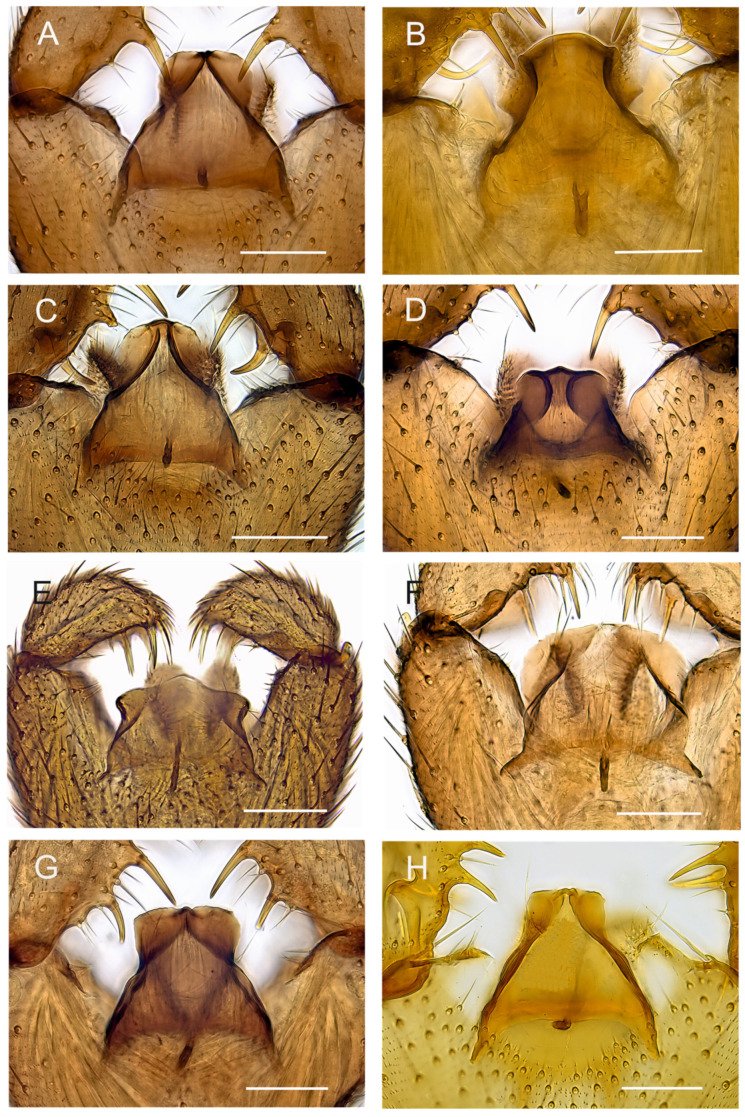
Tegmina, ventral view. (**A**). Holotype MZH GE.1928 of *Spinopygina acerfalx* sp. nov. (**B**). Holotype MZH GE.1932 of *S. aurifera* sp. nov. (**C**). Paratype MZH GE.1936 of *S. camura* sp. nov. (**D**). Holotype MZH GE.1937 of *S. edura* sp. nov. (**E**). Holotype MZH GE.1938 of *S. peltata* sp. nov. (**F**). Holotype MZH GE.1941 of *S. plena* sp. nov. (**G**). Holotype MZH GE.1944 of *S. quadracantha* sp. nov. (**H**). Holotype CNC of *S. uniceps* (Hippa & Vilkamaa, 1994) Scale bars = 0.05 mm.

Abdomen (Figure 4, Figure 5, Figure 6, Figure 7, Figure 8 and Figure 9). Pale brown or yellowish, normal, slender; setae dark, long and strong. Hypopygium brown or yellowish, as abdomen. Intergonocoxal area long or moderate, without lobe(s). Gonocoxa unmodified, longer than gonostylus, gonocoxae fused ventromedially; rather richly setose, setae at ventromedial margin short, medial membrane non-setose; with 1 elongated seta ventrally and dorsally at apicomedial corner. Gonostylus elongated, widest medially and distinctly narrowed towards apex or evenly wide and slightly narrowed towards apex, strongly excavated or at least slightly impressed medially, with lambda-shaped basomedial sclerotization; without apical tooth; with apical, subapical and medial megasetae, or only apical or subapical megasetae present; megasetae strong and with distinct basal bodies, in some species basalmost megaseta distinctly procurved; with some elongated setae medially and apically. Tegmen variable in shape, truncate or slightly curved apically, slightly curved basolaterally, constricted subapically, membranous or with sclerotized rim apically, with flat apicolateral parts; parameres sclerotized, approaching each other and joining to sclerotized rim or ending at apex separately. Apodemes of tegmen and aedeagal apodeme usually short, aedeagal teeth not detectable. Tergite 9 rather narrow, with long and strong setae. Tergite 10 (cercus) normal.

Female unknown.

**Distribution.** USA: California, Oregon; Canada: British Columbia.

**Figure 10:**
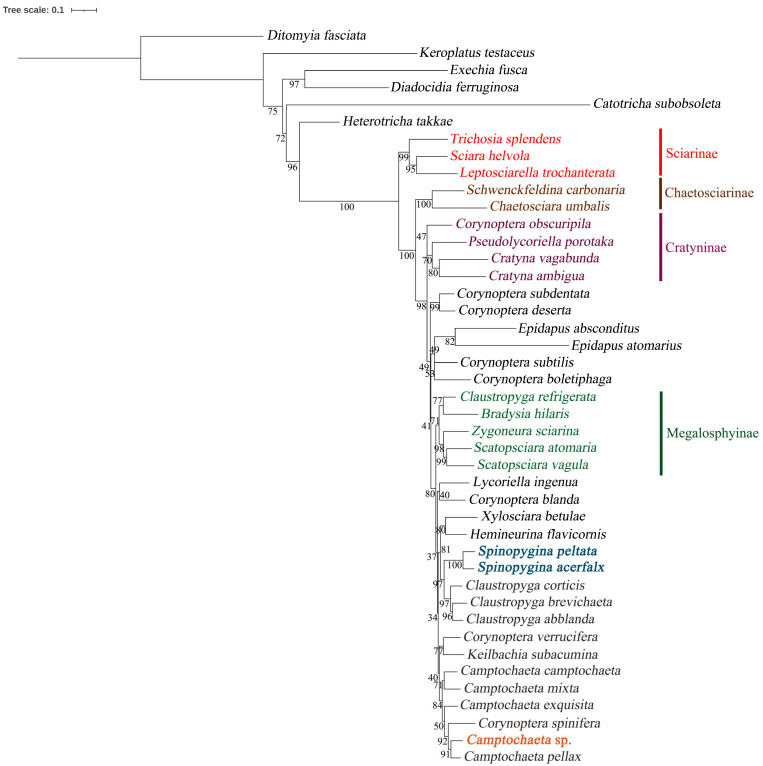
Maximum likelihood hypothesis for relationships among selected taxa of Sciaridae (Diptera) based on DNA sequence data (18S, 28S, 16S, and COI), 4287 characters. Support numbers refer to ultrafast bootstrap values (ufboot) over 50.

### 3.2. Comparative Diagnosis of Spinopygina gen. nov.

Mohrig et al. [13] transferred *Camptochaeta uniceps* Hippa &Vilkamaa, 1994 into the *Corynoptera spinifera* group *sensu* [1]. *Spinopygina* gen. nov. resembles the *C. spinifera* group in having a rather similar lambda-shaped basomedial sclerotized structure of the impressed or excavated gonostylus, in lacking the apical tooth of gonostylus, in having the megasetae with strong basal bodies, in having an apical gonostylar megaseta in all and a strongly curved basal gonostylar megaseta in part of the species. *Spinopygina* differs from the *C. spinifera* group as well as from the *Corynoptera parvula* group sensu [1] in important characters: the long necks of antennal flagellomeres, the 2-segmented maxillary palpus without the sensory pit, the long stalk of the halter, the indistinct, not impressed fore tibial organ, the basoventrally united gonocoxae with distinctly shortened setae at their ventromedial margins and the tegmen with flat apicolateral parts and the apparent lack of the aedeagal teeth; see, e.g., Figure 4, Figure 5 and Figure 30A,B in Hippa & Vilkamaa (1994) [36]. Moreover, *Spinopygina* differs from *Camptochaeta* Hippa &Vilkamaa, 1994 in the sense of [1] in the lack of the apical tooth of the gonostylus in addition to the characters mentioned above.

*Spinopygina* resembles *Claustropyga* Hippa, Vilkamaa & Mohrig, 2003 in having the maxillary palpus 2-segmented (in part of *Claustropyga*), the sensilla scattered on the dorsal side of 1st segment of the maxillary palpus, in having a weakly modified fore tibial organ, in having the medial part of the gonostylus impressed or excavated, in having distinct basal bodies of the gonostylar megasetae and in having the megasetae strong at least in part of the species, in having the gonocoxae united basoventrally with the intergonocoxal area long or at least moderate. *Spinopygina* differs in having the basomedial part of gonostylus impressed with the lambda-shaped sclerotized structure (the impression lacks in *Claustropyga*), in having long flagellomeral necks and stalk of halter and in the lack of the apical tooth of gonostylus (lacks also in one known species of *Claustropyga*). Moreover, even if long, the intergonocoxal area of *Spinopygina* is not lobe-like produced as in most species of *Claustropyga*.

*Spinopygina* resembles *Xylosciara* Tuomikoski, 1960 in having a long intergonocoxal area, in having strong gonostylar megasetae including apical ones and in having a poorly differentiated patch of setae on the fore tibial organ. *Spinopygina* differs most strikingly in having 2-segmented maxillary palpus, longer necks and setae of antennal flagellomeres, in having strong and dark body vestiture and in having a modified tegmen and a strongly hollowed gonostylus.

Some species of *Spinopygina* resemble *Keilbachia* Mohrig, 1987 in having a strong procurved megaseta basally or subbasally on the gonostylus. Some species of *Keilbachia* lack the apical tooth of gonostylus and some species have a strongly modified tegmen reminiscent of that of *Spinopygina*, but *Keilbachia* has a 3-segmented maxillary palpus with a sensory pit on the 1st palpal segment, the fore tibial organ in depression at least in part of the species and it has ventromedially separated gonocoxae.

By the reduced maxillary palpus with sensilla scattered, long antennal flagellomeral bodies and their necks, long and slender legs, the long and oblique mesothoracic katepisternum, the strongly reduced anal lobe of wing and the poorly modified fore tibial organ, *Spinopygina* resembles *Epidapus* Haliday, 1851 and in lacking the apical tooth of gonostylus, especially *Epidapus* (*Pseudoaptagogyna*) *absconditus* group *sensu* [1]. *Epidapus absconditus* (Vimmer, 1926) has, like *Spinopygina*, strong apical-subapical gonostylar megasetae with distinct basal bodies. However, the megasetae of *Epidapus absconditus* are stout, straight and symmetrically narrowed at apex, not long and curved as in *Spinopygina*. The four megasetae of the related *Epidapus quadrispinosus* Mohrig & Mamaev, 1990 are all recurved. Moreover, *Spinopygina* differs from the *E. absconditus* group in having subequal left and right spurs on middle and hindtibia (unequal), in having the intergonocoxal area long with the gonocoxae united basoventrally (short and separate), with shortened setae at the ventromedial margin (equal), in having the tegmen strongly modified and with distinctly sclerotized parameres (simple, laterally and apically roundish and completely membranous tegmen in *Pseudaptagogyna* Vimmer, 1926) and in having the gonostylus impressed or excavated (in *Pseudoaptagogyna* convex medially) and in having (in part of the species) a curved basal megaseta in the gonostylus.

*Spinopygina* differs from all above-mentioned genera in having the tegmen with flat apicolateral parts and in lacking the aedeagal teeth (the teeth not detectable using a compound microscope.

### 3.3. Key to Species of Spinopygina gen. nov.

1. Gonostylus with 5 megasetae …………………………….............. ***S. camura* sp. nov.**
  − Gonostylus with 3–4 megasetae …………………………………………………. 2

2 Gonostylus with 4 megasetae ………………………………………………….………. 3
  − Gonostylus with 3 megasetae …………………………………………………….. 7

3. All megasetae at apical half of gonostylus, tegmen broad apically ….…………….. 4
  − One megaseta at basal half of gonostylus, tegmen narrow apically …………. 5

4. Gonostylus much wider dorsally than ventrally, tegmen with large roundish apicolateral parts ………………………………………………......................***S. plena* sp. nov.**
  − Gonostylus about as wide ventrally and dorsally, tegmen with small acuminate apicolateral parts …………………………………………..........***S. peltata* sp. nov.**

5. Tegmen distinctly constricted medially, roundish apically, parameres joining membraneous apex separately ………………………………................. ***S. aurifera* sp. nov.**
  − Tegmen slightly constricted subapically, truncate apically, parameres joining short sclerotized rim apically ………………………………………………………6

6. Two medial megasetae at ventral margin of gonostylus, basalmost megaseta procurved and with long basal body, arising from medial excavation ………………………………..……………........... ***S. uniceps* (Hippa& Vilkamaa, 1994)**
  − One medial megaseta and basalmost megaseta at ventral margin of gonostylus, basalmost megaseta recurved and with moderate basal body …………… ………………….…………………………………………. ***S. quadracantha* sp. nov.**

7. Tegmen small, its length less than half of length of gonostylus, parameres separately joining broad sclerotized rim apically, apex of tegmen largely sclerotized ………………………………………………….……................................ ***S. edura* sp. nov.**
  − Tegmen large, its length 2/3 of length of gonostylus, parameres united apically, joining narrow sclerotized rim apically, apex of tegmen largely membraneous ……………………………………………………….................... ***S. acerfalx* sp. nov.**

### 3.4. Descriptions of New Species

***Spinopygina acerfalx* sp. nov.**Figure 2B, Figure 3A, Figure 4A and Figure 9A. 

LSID urn:lsid:zoobank.org:act: 008D0F6D-919A-4915-99A8-8AB979A3DAD2.

**Comparative diagnosis.** By its tegmen, *Spinopygina acerfalx* sp. nov. is very similar to *Spinopygina camura* sp. nov., *S. quadracantha* sp. nov. and *S. uniceps* (Hippa & Vilkamaa). All have the tegmen narrowed subapically, with flat apicolateral parts and with the parameres joining a shortly sclerotized rim apically. *Spinopygina acerfalx* is similar to *S. edura* sp. nov. and differs from all above-mentioned species in having only three, not four or five, gonostylar megasetae. Moreover, in the form of the gonostylus and in the arrangement of the gonostylar megasetae *Spinopygina acerfalx* resembles *S. edura*, but the latter is distinct in having the tegmen much smaller and more sclerotized basally and in having the parameres wide apart, joining the broadly sclerotized rim separately at the apex. 

**Etymology.** The name is a Latin noun in apposition, formed from *acer*, sharp, and *falx*, sickle, referring to the sharp and curved gonostylar megasetae.

**Material examined.** Holotype: USA, Oregon, ♂; Benton County; Corvallis; 1460 SW Allen St.; 44.550860° N, 121.270189° W; 4 March–20 April 2015; S. Fitzgerald leg.; Malaise trap; in Euparal; MZH GE.1928. Paratypes: USA, Oregon, 2 ♂♂; Same collection data as for holotype; in Euparal; MZH GE.1929, GE.1945. Oregon, 1 ♂; same collection data as for holotype but 5 April–3 June 2015; in Euparal; MZH GE.1976. Oregon, 2 ♂♂; same collection data as for holotype but 11 December 2014–14 January 2015; used for DNA extraction (samples No. SCI80, SCI81); in Euparal; MZH GE.1978, GE.1979. Oregon, 1 ♂; Benton County; 5 miles up Woods Creek Road from jct Highway 20; 44.544° N, 123.50° W; 25 April–15 May 2014; S. Fitzgerald leg.; Malaise trap; mixed forest; in Euparal; MZH GE.1930. Oregon, 1 ♂; Benton County; Corvallis; 6.4 miles up Woods Creek Road from jct Highway 20; 3 March–12 April 2015; Malaise trap; fir/alder/maple; in Euparal; MZH GE.1946. Oregon, 1 ♂; Coos County; Seven Devils’ Road; 43.3109° N, 124.3484° W; 7 February–15 May 2016; E. Boyd leg; Malaise trap; in Euparal; MZH GE.1931.

**Description.** Male. Head. Face and antenna uniformly brown, maxillary palpus pale brown. Eye bridge 1–2 facets wide. Body of 4th antennal flagellomere 2.60–3.20× as long as subapically wide, the neck longer than wide, the longest setae longer than the width of flagellomere. Face with 5–9 dark long and short setae. Clypeus non-setose or with 1 dark seta. Maxillary palpus with 2 segments; 2nd segment reduced; 1st segment with 1 sharp seta, with large indistinct dorsal patch of sensilla, 2nd segment with 1–5 setae.

Thorax (Figure 2B). Brown; setae dark. Anterior pronotum with 2 setae. Prothoracic episternum with 2–3 setae.

Wing (Figure 3A). Fumose. Length 1.8–2.0 mm. Anal lobe small. Width/length 0.40–0.45. R1/R 0.75–0.85. c/w 0.75–0.80. stM slightly longer than M-fork, bM shorter than or as long as r-m, stCuA shorter or as long as bM. bM and r-m non-setose. Halter yellow, with long stalk.

Legs. Yellow, long. Fore femur slender. Fore tibial organ not impressed or proximally bordered, with small patch of a few setae. Fore tibial spur longer than tibial width. Length of fore basitarsomere/length of fore tibia 0.63.

Abdomen. Pale brown; setae dark, long and strong. Hypopygium (Figure 4A) brown, as abdomen. Intergonocoxal area long, with short setosity. Gonocoxa longer than gonostylus; setae rather short, shorter towards medial margin. Gonostylus elongated, curved, the medial side strongly impressed; with a few elongated setae apically; without apical tooth, with 3 megasetae, 1 apical in ventral position, 1 subapical in dorsal position and 1 medial in ventral position, the megasetae long and strong, slightly recurved, with long basal bodies. Tegmen (Figure 9A) slightly shorter than wide, truncate apically, sharply narrowed subapically; with parameres joining short sclerotized rim apically; with flat apicolateral parts. Aedeagal apodeme short, aedeagal teeth not detectable.

***Spinopygina aurifera*** sp. nov. Figure 1D, Figure 3B, Figure 4B and Figure 9B. 

LSID urn:lsid:zoobank.org:act: B9089867-2FA5-4A03-92E4-5C99E60BD592.

**Comparative diagnosis.** In having the basal megaseta of the gonostylus strongly procurved, *Spinopygina aurifera* sp. nov. resembles *S. camura* sp. nov. and *S. uniceps* (Hippa and Vilkamaa) but by its form of the gonostylus and the tegmen, *S. aurifera* is not very similar to any other known species of the genus: the apicalmost megaseta is shifted to a subapical position and the two medial megasetae are in a lobe-like extension of the ventromedial margin of the gonostylus. The tegmen is characteristic in being strongly constricted at the middle, and in having the apex wide and roundish and in having the flat apicolateral parts small and triangular. *Spinopygina aurifera* differs from all other species of the genus in having a peculiar conical lobe apicodorsally in the gonocoxa.

**Etymology.** The name is a Latin noun in apposition, from the words *auris*, ear, and the suffix -*fer*, bearing, referring to the ear-like apicolateral parts of the tegmen.

**Material examined.** Holotype: USA, Oregon, ♂; Linn County; Hackleman Creek; 0.6 miles E of Tombstone Pass; 44.397501° N, 122.131401° E; 29 June–1 August 2016; S. Fitzgerald leg; Malaise trap; in Euparal; MZH GE.1932. Paratypes: USA, Oregon, 2 ♂♂; same collection data as for holotype; in Euparal; MZH GE.1933 and GE.1934. Oregon, 1 ♂; same collection data as for holotype; in Euparal; MZH GE.1970.

**Description.** Male. Head. Face and antenna uniformly brown, maxillary palpus pale brown. Eye bridge 1–2 facets wide. Body of 4th antennal flagellomere 2.25–2.55× as long as subapically wide, the neck longer than wide, the longest setae longer than the width of flagellomere. Face with 5–7 dark long and short setae. Clypeus non-setose or with 1 dark seta. Maxillary palpus (Figure 1D) with 2 segments; 2nd segment reduced; 1st segment with 1(2) sharp setae, with an indistinct dorsal patch of sensilla, 2nd segment with 3–5 setae.

Thorax. Brown; setae dark. Anterior pronotum with 2 setae. Prothoracic episternum with 2–4 setae.

Wing (Figure 3B). Fumose. Length 1.8–2.0 mm. Width/length 0.40–0.45. Anal lobe small. R1/R 0.75–0.90. c/w 0.70–0.75. stM slightly longer than M-fork, bM longer than r-m, stCuA as long as or shorter than bM. bM and r-m non-setose. Halter yellow, long.

Legs. Yellow, long. Fore femur slender. Fore tibial organ not impressed or proximally bordered, with small patch of a few setae. Fore tibial spur as long as tibial width. Length of fore basitarsomere/length of fore tibia 0.47.

Abdomen. Pale brown; setae dark, long and strong. Hypopygium (Figure 4B) brown, as abdomen. Intergonocoxal area long, with short setosity. Gonocoxa wide, slightly longer than gonostylus; setosity normal, at medial margin shorter. Gonostylus elongated, slightly curved, strongly excavated medially; with a few elongated setae apically and medially; without apical tooth, with 4 megasetae, 1 subapical and 2 medial at ventral margin and 1 basally arising from medial excavation, the megasetae long and strong, nearly straight except the basalmost one procurved, all with basal bodies. Tegmen (Figure 9B) longer than wide, curved apically, constricted laterally, with parameres ending wide apart at apex; with small triangular apicolateral parts. Aedeagal apodeme rather short, aedeagal teeth not detectable.

***Spinopygina camura*** sp. nov. Figure 1C, Figure 5A and Figure 9C. 

LSID urn:lsid:zoobank.org:act: 144A1DDD-712D-4B81-862A-735709641561.

**Comparative diagnosis.** *Spinopygina camura* sp. nov. is distinguished from all other species of the genus in having five instead of three or four gonostylar megasetae, the basalmost of which is strongly procurved (a paratype of *S. quadracantha* has five megasetae on one of its gonostyli) *Spinopygina uniceps* (Hippa and Vilkamaa) and *S. aurifera* sp. nov. also have a procurved basal megaseta on their gonostylus but they have only four megasetae. See also under *S. acerfalx* sp. nov. and *S. quadracantha* sp. nov.

**Etymology.** The name is a Latin adjective, *camura*, curved, referring to the curved basal megaseta of the gonostylus.

**Material examined.** Holotype: USA, Oregon, ♂; Benton County; 6.4 miles up Woods Creek Road from jct Highway 20; 6 March–12 April 2015; S. Fitzgerald leg.; Malaise trap; fir/alder/maple forest; in Euparal; MZH GE.1935. Paratype: USA, Oregon, 1 ♂; same collection data as for holotype; in Euparal; MZH GE.1936.

**Description.** Male. Head. Face and antenna uniformly brown, maxillary palpus pale brown. Eye bridge 2 facets wide. Body of 4th antennal flagellomere (Figure 1C) 2.75–2.90× as long as subapically wide, the neck longer than wide, the longest setae longer than the width of flagellomere. Face with 8–10 dark long and short setae. Clypeus with 1 dark seta. Maxillary palpus with 2 segments; 2nd segment reduced; 1st segment with 1 sharp seta, with an indistinct dorsal patch of sensilla, 2nd segment with 2–3 setae.

Thorax. Brown; setae dark. Anterior pronotum with 2 setae. Prothoracic episternum with 2–4 setae.

Wing. Fumose. Length 2.0 mm. Width/length 0.40–0.45. Anal lobe small. R1/R 0.80. c/w 0.80. stM slightly longer than M-fork, bM shorter than or as long as r-m, stCuA shorter than bM. bM and r-m non-setose. Halter yellow, long.

Legs. Yellow, long. Fore femur slender. Fore tibial organ not impressed or proximally bordered, with small patch of a few setae. Fore tibial spur longer than tibial width. Length of fore basitarsomere/length of fore tibia 0.60.

Abdomen. Pale brown; setae dark, long and strong. Hypopygium (Figure 5A) brown, as abdomen. Intergonocoxal area long, with short setosity. Gonocoxa longer than gonostylus; setae rather short, at medial margin shorter. Gonostylus elongated, curved laterally, strongly impressed medially; with a few elongated setae apically; without apical tooth, with 5 megasetae, 1 apical in ventral position, 1 subapical in dorsal position, 2 medially in ventral position and 1 basally arising from the medial excavation, megasetae long and strong, slightly recurved, the basalmost megaseta strongly procurved, all with long basal bodies. Tegmen (Figure 9C) slightly longer than wide, truncate apically, roundish laterally, with parameres joining to short sclerotized rim apically; with flat apicolateral parts. Aedeagal apodeme short, aedeagal teeth not detectable.

***Spinopygina edura*** sp. nov. Figure 5B and Figure 9D. 

LSID urn:lsid:zoobank.org:act: AE559992-0563-426C-B08C-7CEB91256997.

**Etymology.** The name is a Latin adjective, *edura*, hard, referring to the strongly sclerotized tegmen.

**Comparative diagnosis.** By its gonostylus with a sharp apex and three megasetae *Spinopygina edura* sp. nov. is very similar to *S. acerfalx* sp. nov. although the former has the medial megaseta slightly closer to apex of the gonostylus. *Spinopygina edura* differs from all the species of the genus in its small and more sclerotized tegmen with concave parameres joining a broadly sclerotized rim apically.

**Material examined.** Holotype: USA, Oregon, ♂; Benton County; Corvallis; 1460 SW Allen St.; 44.550860° N, 121.270189° W; ex larva, 19 October 2014, emerged 14 November 2014; S. Fitzgerald; in Euparal; MZH GE.1937.

**Description.** Male. Head. Face and antenna uniformly brown, maxillary palpus pale brown. Eye bridge 1–2 facets wide. Antennae in poor condition in the specimen studied, the neck longer than wide. Face with 10 dark long and short setae. Clypeus with 1 dark seta. Maxillary palpus with 2 segments; 2nd segment reduced; 1st segment with 1 sharp seta, with an indistinct dorsal patch of sensilla, 2nd segment with 3–4 setae.

Thorax. Brown; setae dark. Anterior pronotum with 1 seta. Prothoracic episternum with 3 setae.

Wing. Fumose. Length 2.2 mm. Width/length 0.40. Anal lobe small. R1/R 0.70. c/w 0.80. stM slightly longer than M-fork, bM shorter than r-m, stCuA shorter than bM. bM and r-m non-setose. Halter yellow, long.

Legs. Yellow, long. Fore femur slender. Fore tibial organ not impressed or proximally bordered, with small patch of a few setae. Fore tibial spur slightly longer than tibial width. Length of fore basitarsomere/length of fore tibia 0.60.

Abdomen. Pale brown; setae dark, long and strong. Hypopygium (Figure 5B) brown, as abdomen. Intergonocoxal area long, with short setosity. Gonocoxa longer than gonostylus; setae rather short, at medial margin shorter. Gonostylus elongated, curved, the medial side impressed; with a few elongated setae apically; without apical tooth, with 3 megasetae, 1 at very apex in dorsal position, 1 subapically in ventral position and 1 medially in ventral position; the megasetae long and strong, slightly recurved, with long basal bodies. Tegmen (Figure 9D) slightly shorter than wide, truncate apically, concave laterally, with strong basolateral sclerotizations, with concave parameres joining separately broad sclerotized rim apically; with large flat apicolateral parts. Aedeagal apodeme short, aedeagal teeth not detectable.

***Spinopygina peltata*** sp. nov. Figure 1B, Figure 3D, Figure 6A and Figure 9E. 

LSID urn:lsid:zoobank.org:act: E7434F57-4965-4501-B26C-3A0C49D11302.

**Comparative diagnosis.** In having four gonostylar megasetae all at the apical half of half of the gonostylus and in having a wide tegmen, *Spinopygina peltata* sp. nov. resembles *S. plena* sp. nov. but differs in having the tegmen constricted subapically and with acuminate flat apicolateral parts. Moreover, *Spinopygina plena* has the dorsal side of its gonostylus greatly enlarged.

**Etymology.** The name is formed from the Latin word *pelta*, shield, referring to the wide tegmen.

Material examined. Holotype: USA, Oregon, ♂; Benton County; Corvallis; 1460 SW Allen St.; 44.550860° N, 121.270189° W; 4 March–20 April 2015; S. Fitzgerald leg.; Malaise trap; in Euparal; MZH GE.1938. Paratypes: USA, Oregon, 6 ♂♂; same collection data as for holotype; MZH GE.1939, GE.1947–GE.1951. Oregon, 1 ♂; same collection data; in Euparal; MZH GE.1971. Oregon, 1 ♂; same collection data but 11 December 2014–15 January 2015; in Euparal; MZH GE.1980. Oregon, 2 ♂♂; same data as previous, used for DNA extraction (samples No. SCI82, SCI83), MZH GE.1973, GE.1974. Oregon, 1 ♂; Coos County; Seven Devils’ Road; 43.3109° N, 124.3484° W; 7 February–15 May 2016; E. Boyd leg; Malaise trap; in Euparal; MZH GE.1940. Oregon, 1 ♂; Coos County; Charleston; Seven Devils’ Road; 43.3132° N, 124.3485° W; 7 February–15 May 2016; S. Fitzgerald leg; Malaise trap; in Euparal; MZH GE.1972.

**Description.** Male. Head. Face brown, darker than antenna, maxillary palpus pale brown. Eye bridge 2 facets wide. Body of 4th antennal flagellomere 2.75–3.2× (Figure 1B) as long as subapically wide, the neck longer than wide, the longest setae longer than the width of flagellomere. Face with 5–9 dark long and short setae. Clypeus with 1 dark seta. Maxillary palpus with 2 segments; 2nd segment reduced; 1st segment with 1–2 sharp setae, with an indistinct dorsal patch of sensilla, 2nd segment with 2 setae.

Thorax. Brown; setae dark. Anterior pronotum with 1–2 setae. Prothoracic episternum with 2–5 setae.

Wing. Fumose. Length 1.7–2.1 mm. Width/length 0.40–0.45. Anal lobe small. R1/R 0.80–1.15. c/w 0.75–0.90. stM slightly longer than M-fork, bM longer than or as long as r-m, stCuA shorter than r-m. bM and r-m non-setose. Halter yellow, long.

Legs (Figure 3D). Yellow, long. Fore femur slender. Fore tibial organ not impressed or proximally bordered, with small patch of a few setae. Fore tibial spur longer than tibial width. Length of fore basitarsomere/length of fore tibia 0.58.

Abdomen. Pale brown; setae dark, long and strong. Hypopygium (Figure 6A) brown, as abdomen. Intergonocoxal area long, with short setosity. Gonocoxa rather narrow, much longer than gonostylus; setae rather short, at medial margin shorter. Gonostylus elongated, rather straight, the medial side impressed; with a few elongated setae apically; without apical tooth, with 4 megasetae, 1 at apex in dorsal position, 1 subapically in ventral position and 2 slightly more mediad in dorsal and ventral positions, the megasetae long and strong, nearly straight, with short basal bodies. Tegmen (Figure 9E) shorter than wide, hyaline and slightly produced apically, roundish basolaterally and slightly constricted subapically, with parameres ending separately wide apart at apex; with flat apicolateral parts. Aedeagal apodeme long, aedeagal teeth not detectable.

***Spinopygina plena* sp. nov.**Figure 1A,E, Figure 3E, Figure 6B and Figure 9F. 

LSID urn:lsid:zoobank.org:act: 8F5CAF1D-108C-4008-A048-35CA415C8E19.

**Comparative diagnosis.** *Spinopygina plena* sp. nov. resembles *S. peltata* sp. nov., see under the latter.

**Etymology.** The name is a Latin adjective, *plena*, plump, referring to the tumid gonostylus.

**Material examined.** Holotype: USA, California, ♂; Los Angeles County; 9 km N of La Canada; 34°25′ N, 118°19′ W; 900 m; 14 December 1994; B.V. Brown leg.; Malaise trap; ravine with oak forest; in Euparal; MZH GE.1941. Paratypes: USA, California, 2 ♂♂; same collection data as for holotype; in Euparal; MZH GE.1942, GE.1943. California, 1 ♂; same collection data as for holotype but 20 January–2 February 1995; B.V. Brown leg.; in Euparal; MZH GE.1981.

**Description.** Male. Head (Figure 1A). Face brown, darker than antenna, maxillary palpus pale brown. Eye bridge 2 facets wide. Body of 4th antennal flagellomere 2.3–2.9× as long as subapically wide, the neck longer than wide, the longest setae longer than the width of flagellomere. Face with 6–10 dark long and short setae. Clypeus non-setose or with 1 dark seta. Maxillary palpus (Figure 1E) with 2 segments; 2nd segment reduced; 1st segment with 1–2 sharp setae, with an indistinct dorsal patch of sensilla, 2nd segment with 1–2 setae.

Thorax. Brown; setae dark. Anterior pronotum with 2–4 setae. Prothoracic episternum with 3–5 setae.

Wing. Fumose. Length 1.6–1.7 mm. Width/length 0.40. Anal lobe small. R1/R 0.80–0.95. c/w 0.75–0.80. stM slightly longer than M-fork, bM longer than r-m, stCuA shorter than r-m. bM and r-m non-setose. Halter yellow, long.

Legs. Yellow, long. Fore femur slender. Fore tibial organ (Figure 3E) not impressed or proximally bordered, with indistinct patch of a few setae. Fore tibial spur longer than tibial width. Length of fore basitarsomere/length of fore tibia 0.50.

Abdomen. Pale brown; setae dark, long and strong. Hypopygium (Figure 6B) brown, as abdomen. Intergonocoxal area long, with short setosity. Gonocoxa longer than gonostylus; setosity rather sparse, at medial margin shorter. Gonostylus wide, curved laterally, strongly impressed medially, distinctly lobe-like produced dorsomedially, with a few elongated setae apically without apical tooth, with 4 megasetae, 1 at the very apex, 2 subapically, 1 medially, the megasetae rather long and strong, nearly straight, with distinct basal bodies. Tegmen (Figure 9F) slightly shorter than wide, truncate apically, roundish laterally, with lateral sclerotizations (parameres) joining wide apart at apex; with large flat apicolateral parts. Aedeagal apodeme short, aedeagal teeth not detectable.

***Spinopygina quadracantha* sp. nov.**Figure 7 and Figure 9G.

LSID urn:lsid:zoobank.org:act: E8ADE6BD-FF9F-4F09-931A-3DAD514FDCEA.

**Comparative diagnosis.** In having all gonostylar megasetae long and slightly recurved, *Spinopygina quadracantha* sp. nov. resembles *S. acerfalx* sp. nov. and *S. edura* sp. nov. but differs in having four instead of three megasetae. However, two of the studied specimens have an additional procurved megaseta at the basal part in one of their gonostyli (Figure 7B). These gonostyli with 5 megasetae are difficult to distinguish from those of *Spinopygina camura* sp. nov. but the tegmen of *S. quadracantha* is bigger with larger apicolateral flat parts. A large tegmen with convex parameres joining into a short sclerotized rim apically is fairly similar to that of *S. acerfalx* and differs distinctly from that of *S. edura* sp. nov. See also under the latter.

**Etymology.** The name is a Latin noun in apposition, from the words *quadrus*, four, and *acantha*, spine, referring to the four gonostylar megasetae.

**Material examined.** Holotype: Canada, British Columbia, ♂; Vancouver Island; Rocky Point; 19 March 1995; N. Winchester leg.; Malaise trap; in Euparal; MZH GE.1944.

Paratypes: Canada, British Columbia, 7 ♂♂; same collection data as for holotype but 29 October 1995; MZH GE.1952 to GE.1958. British Columbia, 2 ♂♂; same collection data as previous; in Euparal, MZH GE.1974, GE.1975. British Columbia, 2 ♂♂; same collection data as previous but 15 January 1995; in Euparal; MZH GE.1959, GE.1960.

**Description.** Male. Head. Face and antenna uniformly brown, maxillary palpus pale brown. Eye bridge 2 facets wide. Body of 4th antennal flagellomere 2.40–3× as long as subapically wide, the neck longer than wide, the longest setae longer than the width of flagellomere. Face with 4–10 dark long and short setae. Clypeus with 1 dark seta. Maxillary palpus with 2 segments; 2nd segment reduced; 1st segment with 1–2 sharp setae, with an indistinct dorsal patch of sensilla, 2nd segment with 1–3 setae.

Thorax. Brown; setae dark. Anterior pronotum with 2 setae. Prothoracic episternum with 3 setae.

Wing. Fumose. Length 1.8–2.1 mm. Width/length 0.40–0.45. Anal lobe small. R1/R 0.85–1.0. c/w 0.70–0.90. stM slightly longer, as long as or shorter than M-fork, bM longer or shorter than r-m, stCuA shortest. bM and r-m non-setose. Halter yellow, long.

Legs. Yellow, long. Fore femur slender. Fore tibial organ with weak vestiture, forming an indistinct patch of few setae. Fore tibial spur longer than tibial width. Length of fore basitarsomere/length of fore tibia 0.60.

Abdomen. Pale brown; setae dark, long and strong. Hypopygium (Figure 7) brown, as abdomen. Intergonocoxal area long, with short setosity. Gonocoxa longer than gonostylus; setae rather short, at medial margin shorter. Gonostylus elongated, curved, strongly impressed medially; with a few elongated setae apically and medially; without apical tooth, with 4 (rarely 5) megasetae, 1 at the very apex, 1 subapically and 2 medially wide apart, the megasetae long and strong, slightly recurved, with long basal bodies. Tegmen (Figure 9D) longer than wide, truncate apically, constricted laterally, with parameres joining to short sclerotized rim apically; with large flat apicolateral parts. Aedeagal apodeme short, aedeagal teeth not detectable.

***Spinopygina uniceps*** (Hippa & Vilkamaa, 1994) comb. nov. Figure 2A, Figure 8 and Figure 9H. *Camptochaeta uniceps* Hippa & Vilkamaa, 1994: 56, Figure 30A,B.

*Corynoptera uniceps* Mohrig et al. (2013): 195.

**Short redescription.** Male. Wing length 1.6–2.2 mm (Figure 2A). 1st segment of maxillary palpus with 1 seta, 2nd segment of with 2–5 setae. Gonostylus (Figure 8) widest medially, strongly narrowed towards apex, strongly excavated medially, apex with rather short apical megaseta; with two medial megasetae at dorsomedial margin, basal megaseta with long basal body arising from medial excavation. Tegmen (Figure 9H) with flat apicolateral parts, parameres joining short sclerotized rim apically.

**Comparative diagnosis.** By its form of gonostylus with four megasetae and by the tegmen with flat apicolateral parts and the parameres joining a short apical rim, *Spinopygina uniceps* (Hippa & Vilkamaa, 1994) most resembles *S. quadracantha* sp. nov. but differs in having a shorter apical megaseta of the gonostylus, in having the basal megaseta distinctly procurved and with a long basal body and placed in the medial excavation instead at the ventromedial margin of the gonostylus. *Spinopygina uniceps* has the apex of tegmen narrower and with smaller flat apicolateral parts (Figure 7 and Figure 8). See also under *Spinopygina acerfalx*.

**Material examined.** Holotype: Canada, British Columbia, ♂; Vancouver Island; Upper Carmanah Valley; 30 July 1991; N. Winchester leg.; Malaise trap; forest floor; in Euparal; CNC. Paratypes. Canada, British Columbia, 3 ♂♂; same collection data as for holotype; in Euparal; MZH GE.1116 to GE.1118. British Columbia, 1 ♂; same collection data as for holotype; in Euparal; RBCM.

**New material.** Canada, British Columbia, 3 ♂♂; Vancouver Island; Upper Carmanah Valley; 10–29 September 1991; N. Winchester leg; Malaise trap, forest floor; in Euparal; MZH GE.1961 to GE.1963. British Columbia, 5 ♂♂; same data as previous but 17–26 October 1991; in Euparal; MZH GE.1964 to GE.1968. British Columbia, 1 ♂; same collection data as previous; in ethanol; MZH GE.1969. British Columbia, 1 ♂; same collection data as previous; in Euparal; GE.1977.

### 3.5. Phylogeny

In general, the present hypothesis (Figure 10), based on the same gene markers, is similar to the earlier published phylogenies [9,10,12]. The proposed subfamilies Sciarinae, Chaetosciarinae, Cratyninae and Megalosphyinae appear with good supports, as well as the polyphyletic *Pseudolycoriella* group of genera.

In this study, *Spinopygina* nested with *Claustropyga* as its sister group. As in the analysis of [12], *Claustropyga* in its original sense [3] was not monophyletic, *C. refrigerata* (Lengersdorf, 1930), in the earlier morphological cladistic analysis [3] appearing as the sister group of all other species of *Claustropyga*, now appeared in the Megalosphyinae clade. Moreover, *Pseudolycoriella porotaka* nested in the Cratyninae clade, although with low support. The placement of this genus was different also in the previous molecular analyses [9,12]. Morphologically, *Pseudolycoriella* is not very similar to any other genus of Sciaridae.

The former placement [13] of *S. uniceps* into the *Corynoptera spinifera* group proposed by Menzel and Mohrig (2000) [1] is not supported, and that group appears as polyphyletic. Interestingly, the two analyzed species of the group, *C. spinifera* Tuomikoski, 1960 and *C. verrucifera* (Lengersdorf, 1952), included in *Camptochaeta* [36] and subsequently transferred into the *C. spinifera* group [1], in the present hypothesis appear in the *Camptochaeta* clade with strong support. Moreover, the present hypothesis supports the close relationship between *Keilbachia* and *Camptochaeta*. The consequent changes in the classification as well as the description of the new species of *Camptochaeta* with an exceptional tegmen will be done in another publication (Vilkamaa et al. in prep.) Morphological similarities and differences in *Spinopygina* to *Claustropyga*, *Corynoptera spinifera* group and other relevant taxa are discussed under Comparative diagnosis of *Spinopygina*.

## 4. Discussion

*Spinopygina* gen. nov. has the structure of its tegmen as a unique character. By the combination of this and other characters, although the latter shared with many other genera, the genus is easy to identify.

*Spinopygina* shares quite a few morphological characters with many other genera, and a hypothesis of its phylogenetic position and of the sister group relationship between *Spinopygina* and *Claustropyga* were only possible by analyzing molecular characters. Although the molecular data were available from only two of the eight species, these species represent two different morphological types of *Spinopygina*, and the monophyly of the eight known species is well founded.

Judging from the few collection localities of the material for the present study, it is highly probable that more species of the new genus will be found in the western Nearctic region.

Regarding the whole sciarid fauna, the Nearctic region is far less known than the Palaearctic fauna [1,13] and it is possible that more new genera remain to be discovered in the region, especially in the less studied western parts.

## Figures and Tables

**Table 1:** List of specimens used for the phylogenetic analysis, with GenBank accession numbers. The voucher codes are stated only in the newly sequenced species.

Species	Voucher	16S	28S	18S	COI
*Bradysia hilaris*		JQ613922	JQ613726	JQ613630	JQ613822
*Camptochaeta camptochaeta*		JQ613970	JQ613773	JQ613675	JQ613870
*Camptochaeta exquisita*	SCI84	OQ024861	OQ024849	OQ024870	OQ024762
*Camptochaeta mixta*	SCI89	OQ024864	OQ024852	OQ024873	OQ024765
*Camptochaeta pellax*	SCI85	OQ024862	OQ024850	OQ024871	OQ024763
*Camptochaeta* sp.	SCI87	OQ024863	OQ024851	OQ024872	OQ024764
*Catotricha subobsoleta*		MG554124	MG554155	KP288784	KT316873
*Chaetosciara umbalis*		JQ613905	JQ613709	JQ613613	JQ613805
*Claustropyga abblanda*		MG554121	MG554146	MG554134	MG554165
*Claustropyga brevichaeta*	SCI113	OQ024865	OQ024853	OQ024874	OQ024766
*Claustropyga corticis*	SCI122	-	-	-	OQ024769
	SCI123	OQ024867	OQ024856	OQ024877	-
*Claustropyga refrigerata*		N/A	KU949253	N/A	KU923133
*Corynoptera blanda*		JQ613965	JQ613768	JQ613670	JQ613865
*Corynoptera boletiphaga*		JQ613974	JQ613777	JQ613679	JQ613874
*Corynoptera deserta*		KU949092	KU949259	N/A	KU923138
*Corynoptera obscuripila*		-	-	-	MZ625500
*Corynoptera spinifera*	BOLD	N/A	N/A	N/A	SCINO533-15
*Corynoptera subdentata*	SCI118	OQ024866	OQ024855	OQ024876	OQ024768
*Corynoptera subtilis*		JQ613978	JQ613781	JQ613682	JQ613878
*Corynoptera verrucifera*	SCI114	N/A	OQ024854	OQ024875	OQ024767
*Cratyna vagabunda*		JQ613968	JQ613771	JQ613673	JQ613968
*Cratyna ambigua*		JQ613929	JQ613733	JQ613637	JQ613829
*Diadocidia ferruginosa*		MG554126	MG554157	KP288786	KC435634
*Ditomyia fasciata*		MG554125	MG554156	MG554141	MG554168
*Epidapus absconditus*		N/A	KU949293	N/A	KU923211
*Epidapus atomarius*		JQ613971	JQ613774	JQ613676	JQ613871
*Exechia fusca*		MG554126	MG554158	MG684611	MG684785
*Hemineurina flavicornis*		JQ613885	JQ613689	JQ613593	JQ613786
*Heterotricha takkae*		MG554128	MG554159	MG684612	MG684786
*Keilbachia subacumina*		JQ613951	JQ613755	N/A	JQ613851
*Keroplatus testaceus*		MG554129	MG554160	KP288746	KT316834
*Leptosciarella trochanterata*		JQ613941	JQ613745	JQ613648	JQ613841
*Lycoriella ingenua*		JQ613901	JQ613705	JQ613609	JQ613802
*Pseudolycoriella porotaka*		MK906478	MK906568	N/A	MK906375
*Scatopsciara atomaria*		JQ613973	JQ613776	JQ613678	JQ613873
*Scatopsciara vagula*		JQ613967	JQ613770	JQ613672	JQ613867
*Schwenckfeldina carbonaria*		MG554120	MG554145	MG554133	MG554164
*Sciara helvola*		JQ613911	JQ613715	JQ613619	JQ613811
*Spinopygina acerfalx*	SCI80	OQ024859	OQ024847	OQ024868	OQ024760
*Spinopygina peltata*	SCI82	OQ024860	OQ024848	OQ024869	OQ024761
*Trichosia splendens*		JQ613969	JQ613772	JQ613674	JQ613869
*Xylosciara betulae*		JQ613963	JQ613766	JQ613668	JQ613863
*Zygoneura sciarina*		JQ613909	JQ613713	JQ613617	JQ613809

## Data Availability

The data presented in this study are available on request from the corresponding author. The data are not publicly available due to ongoing research.
